# Supplementary material for: The first transmembrane region of complement component-9 acts as a brake on its self-assembly
Source: Nat Commun. 2018 Aug 15;9:3266. doi: 10.1038/s41467-018-05717-0 (PMC6093860; doi:10.1038/s41467-018-05717-0)
Supplement: Supplementary file 1 — Supplementary information [file 41467_2018_5717_MOESM1_ESM.pdf]

The first transmembrane region of Complement component-9 acts as a brake on its self-assembly

Spicer *et al.*

SUPPLEMENTARY INFORMATION

Supplementary Table 1 Intermolecular contacts between adjacent molecules of polyC9. Chain A and B are the elongating and binding molecules, respectively.

Intermolecular hydrophobic interactions (< 5 Å)

Residue No	Residue	Chain	Residue No	Residue	Chain
60	ALA	A	454	PRO	B
60	ALA	A	456	TYR	B
146	PRO	A	286	VAL	B
147	LEU	A	182	ILE	B
379	PHE	A	214	PHE	B
379	PHE	A	216	ALA	B
392	ALA	A	203	PHE	B
393	VAL	A	200	ILE	B
393	VAL	A	202	ALA	B
395	ILE	A	200	ILE	B
404	VAL	A	191	PHE	B
417	PHE	A	280	ILE	B
417	PHE	A	434	PHE	B
417	PHE	A	435	VAL	B
417	PHE	A	438	ALA	B

Intermolecular Cation-Pi interactions (< 6 Å)

107	PHE	A	122	ARG	B
133	ARG	A	171	TYR	B
522	LYS	A	196	TYR	B
420	LYS	A	434	PHE	B

Inter main-chain hydrogen bonds

Residue No	Residue	Chain	Atom	Residue No	Residue	Chain	Atom
380	A	ASN	N	215	B	ASN	O
382	A	ASP	N	213	B	ASN	O
384	A	CYS	N	211	B	THR	O
386	A	LYS	N	209	B	GLU	O
388	A	GLY	N	207	B	ILE	O
390	A	GLY	N	205	B	SER	O
392	A	ALA	N	203	B	PHE	O
394	A	ASN	N	201	B	GLU	O
396	A	THR	N	199	B	GLN	O
398	A	GLU	N	197	B	GLU	O
400	A	LEU	N	195	B	HIS	O
402	A	ASP	N	193	B	THR	O
404	A	VAL	N	191	B	PHE	O
406	A	SER	N	189	B	LYS	O
408	A	ILE	N	187	B	GLY	O
187	B	GLY	N	408	A	ILE	O
189	B	LYS	N	406	A	SER	O
191	B	PHE	N	404	A	VAL	O

193	B	THR	N	402	A	ASP	O
195	B	HIS	N	400	A	LEU	O
197	B	GLU	N	398	A	GLU	O
199	B	GLN	N	396	A	THR	O
201	B	GLU	N	394	A	ASN	O
203	B	PHE	N	392	A	ALA	O
205	B	SER	N	390	A	GLY	O
207	B	ILE	N	388	A	GLY	O
209	B	GLU	N	386	A	LYS	O
211	B	THR	N	384	A	CYS	O
213	B	ASN	N	382	A	ASP	O
215	B	ASN	N	380	A	ASN	O
**Main-chain and side-chain hydrogen bonds**							
59	A	ASP	OD2	452	B	LEU	O
59	A	ASP	OD2	452	B	LEU	O
66	A	GLN	OE1	461	B	VAL	O
66	A	GLN	OE1	461	B	VAL	O
147	A	LEU	N	180	B	SER	OG
382	A	ASP	OD1	213	B	ASN	O
382	A	ASP	OD1	213	B	ASN	O
384	A	CYS	N	211	B	THR	OG1
399	A	ASN	OD1	195	B	HIS	O
399	A	ASN	OD1	195	B	HIS	O
403	A	ASP	OD1	191	B	PHE	O
403	A	ASP	OD1	191	B	PHE	O
425	A	ARG	NH2	429	B	ILE	O
425	A	ARG	NH2	429	B	ILE	O
164	B	ARG	NH1	443	A	ASP	O
164	B	ARG	NH1	443	A	ASP	O
185	B	THR	OG1	411	A	GLY	O
190	B	ASN	OD1	404	A	VAL	O
190	B	ASN	OD1	404	A	VAL	O
211	B	THR	OG1	384	A	CYS	O
289	B	ASN	ND2	316	A	ALA	O
289	B	ASN	ND2	316	A	ALA	O
487	B	ARG	NH2	53	A	ASN	O
487	B	ARG	NH2	53	A	ASN	O
**Inter side-chain hydrogen bonds**							
320	A	THR	OG1	289	B	ASN	ND2
387	A	ARG	NH1	208	B	GLN	NE2
387	A	ARG	NH1	208	B	GLN	NE2
394	A	ASN	ND2	201	B	GLU	OE1
394	A	ASN	ND2	201	B	GLU	OE1
189	B	LYS	NZ	421	A	GLU	OE1
189	B	LYS	NZ	421	A	GLU	OE2
201	B	GLU	OE1	394	A	ASN	ND2
201	B	GLU	OE1	394	A	ASN	ND2
288	B	ARG	NH2	301	A	ASP	OD2

288	B	ARG	NH2	301	A	ASP	OD2
289	B	ASN	ND2	320	A	THR	OG1
289	B	ASN	ND2	320	A	THR	OG1
290	B	ARG	NE	313	A	GLU	OE1
487	B	ARG	NH1	19	A	HIS	NE2
487	B	ARG	NH1	19	A	HIS	NE2
487	B	ARG	NH2	19	A	HIS	NE2
487	B	ARG	NH2	19	A	HIS	NE2

Supplementary Table 2 Substructure movement of poly C9 in comparison with monomeric C9*.

Fragment	Residue range[3]	Structure components	rmsd Å	Translation Å	Number of residues	Number conserved
RB1[1]	286-329	MACPF	0.917	-	65	52
	452-472					
RB2[1]	80-110	LDL	1.082	1.770	73	60
	119-134					
	151-166	MACPF				
	172-180					
TSP[1]	18-67	TSP1	0.849	2.551	50	37
Helix-EGF[1]	474-520	EGF	0.864	3.013	47	30
TSP-EGF[1]	18-24	TSP1	0.864	2.533	66	43
	47-58					
	474-520	EGF				
HTH-2[2]	408-413	HTH helix 2	0.524	-	23	16
	431-447					
HTH-1[2]	415-424	HTH helix 1	0.334	5.205	10	6

*Analysis was performed on human poly C9 and murine monomer C9 and their respective movements from soluble to pore transition.

[1]The alignment of RB1 of monomer C9 (see residue range in Table) and poly C9 was used as a probe to determine the translation to the RB2, TSP, Helix-EGF and TSP-EGF substructures.

[2]The alignment of the HTH-2 regions was used as a probe to determine the translation of the HTH-1 helix.

[3]Amino acid numbering of the rigid body fragments is based on the sequence of human C9.

Supplementary Table 3 Oligonucleotide primers for cloning and site-directed mutagenesis

PRIMER	SEQUENCE
**oBS86**	5'-GAGGCGGCCCAGCCGGCCCAGTACACGACCAGTTATGACCC-3'
**oBS101**	5'-ACTTACCAACTATGTTTGTCATATTCTTCAAAG-3'
**oBS102**	5'-GAAGAATATGACAAACATAGTTGGTAAGTTTC-3'
**oBS105**	5'-CATAGATGATGTTTGTTCACTCATAAGAGGTGG-3'
**oBS106**	5'-CTCTTATGAGTGAACAAACATCATCTATGAG-3'
**BGH**	5'-TAGAAGGCACAGTCGAGG-3'

Supplementary Table 4 Data collection, for MIRAS phasing and refinement statistics*

	Native	Crystal 1 (Tantalum bromide)	Crystal 2 (Uranyl format)
**Data collection**			
Space group	P 2_1 2 2_1	P 2_1 2 2_1	P 2_1 2 2_1
Cell dimensions			
a, *b*, *c* (Å)	52.91 148.87 165.78	52.91 148.29 165.43	52.59 149.21 165.83
α, β, γ (°)	90.00 90.00 90.00	90.00 90.00 90.00	90.00 90.00 90.00
Wavelength (eV)	10300	10300	10300
Resolution (Å)	2.2	2.3	2.5
R_{merge}	0.075 (1.359)	0.09 (2.182)	0.1 (1.341)
R_{pim}	0.023 (0.514)	0.02 (0.586)	0.023 (0.323)
$I / \sigma I$	16.8 (1.4)	1 (0.59)	20 (2)
Completeness (%)	98.7 (97.3)	99.1 (95.5)	99.8 (98.5)
Redundancy	12.1 (8.6)	20.2 (15.1)	21.8 (18.5)
**Refinement**			
Resolution (Å)		2.20	
No. reflections (work/free)		66058/3369	
R_{work} / R_{free}		20.91/25.02	
No. atoms			
Protein (Chain A/B)		3632/3528	
Ligand/ion (Chain X/Y/Z)		29/29/1	
Water		335	
B-factors			
Protein (Chain A/B)		72.43/62.66	
Ligand/ion (Chain X/Y/Z)		91.84/75.47/99.17	
Water		63.15	
R.m.s deviations			
Bond lengths (Å)		0.008	
Bond angles (°)		0.98	

*Values in parentheses are for highest-resolution shell.

Supplementary Table 5 Data collection for Zinc Verification *

	Low Energy	High Energy
**Data collection**		
Space group	P 2_1 2 2_1	P 2_1 2 2_1
Cell dimensions		
a, *b*, *c* (Å)	53.46 148.86 165.03	53.56 149.06 165.37
α, β, γ (°)	90.00 90.00 90.00	90.00 90.00 90.00
Wavelength (eV)	9643.9	9674.0
Resolution (Å)	2.48	2.6
R_{merge}	0.116 (1.849)	0.171 (3.125)
R_{pim}	0.02 (0.453)	0.042 (0.764)
$I / \sigma I$	17.9 (1.7)	13.7 (0.9)
Completeness (%)	99.8 (98.4)	100 (100)
Redundancy	18.0 (18.1)	18 (18.5)

*Values in parentheses are for highest-resolution shell.

Supplementary Table 6 Cryo-EM data collection, refinement and validation statistics

	PolyC9 (EMDB-7773) (PDB id 6DLW)
**Data collection and processing**	
Magnification	75,000
Voltage (kV)	300
Electron exposure (e–/Å^2)	46.4
Defocus range (μm)	- 0.5 to -3.0
Pixel size (Å)	1.06
Symmetry imposed	C22
Initial particle images (no.)	220,000
Final particle images (no.)	52,000
Map resolution (Å)	3.9
FSC threshold	0.143
Map resolution range (Å)	3.2 – 4.5
**Refinement**	
Initial model used (PDB code)	6CXO
Model resolution (Å)	3.9
FSC threshold	0.143
Model resolution range (Å)	3.2 – 4.5
Map sharpening *B* factor (Å^2)	-180
Model composition	
Non-hydrogen atoms	3,630
Protein residues	460
Ligands	25
B factors (Å^2)	
Protein	-180
Ligand	-180
Validation	
MolProbity score	1.80 (85th percentile)
Clashscore	4.87 (94th percentile)
Poor rotamers (%)	0.25%
Ramachandran plot	
Favored (%)	90.17
Allowed (%)	9.83
Disallowed (%)	0.0

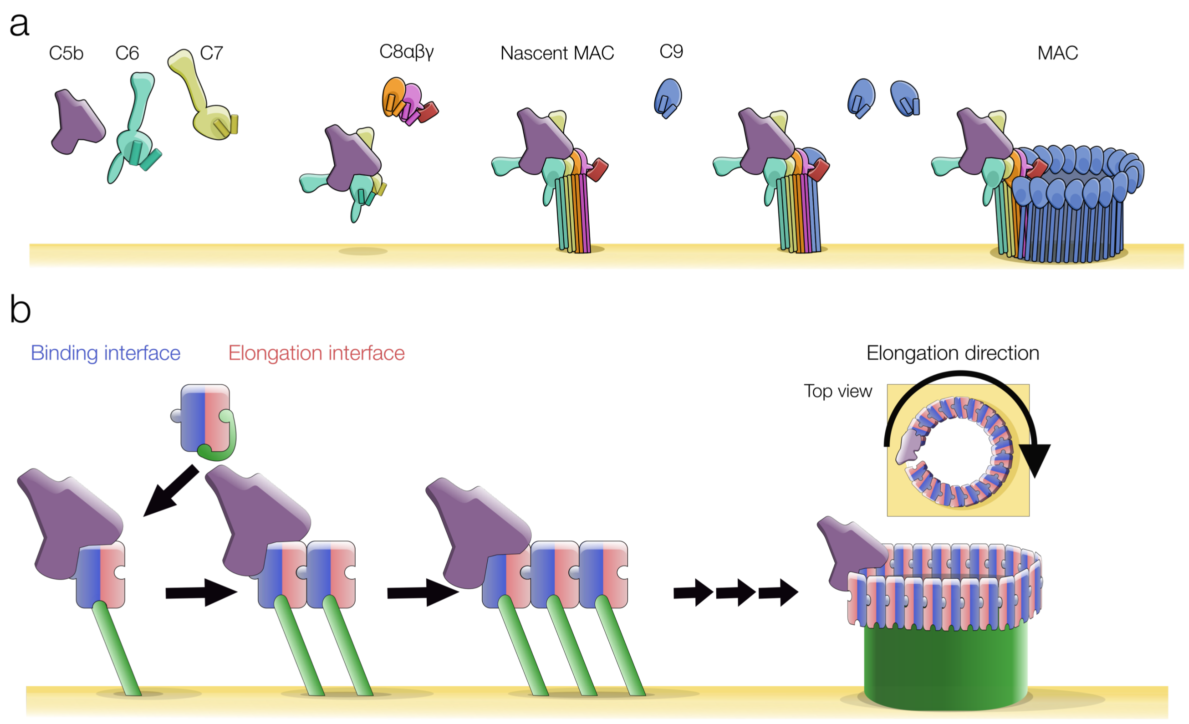

Supplementary Figure 1 a) Schematic diagram of MAC assembly. Soluble C5b forms a metastable complex with C6 and C7 (to C5b7) that superficially anchors to membranes. The C5b7 recruits C8 (αβγ) to form the nascent MAC C5b8. Seventeen to nineteen molecules of the C9 component then oligomerize with the nascent MAC forming a large membrane spanning β-barrel. The C6, C7, C8α and C8β and C9 components each contribute two β-hairpins to the barrel shown in the soluble components as cylinders and the membrane bound form as long sticks. **b**) Schematic illustration of unidirectional assembly of C9. Each component of the MAC contains a binding surface and an elongation surface. Once an individual component is associated with the nascent MAC, its elongation surface is activated (presumably *via* a conformational change) such that it can now interact with the binding surface of the next soluble component to join the complex.

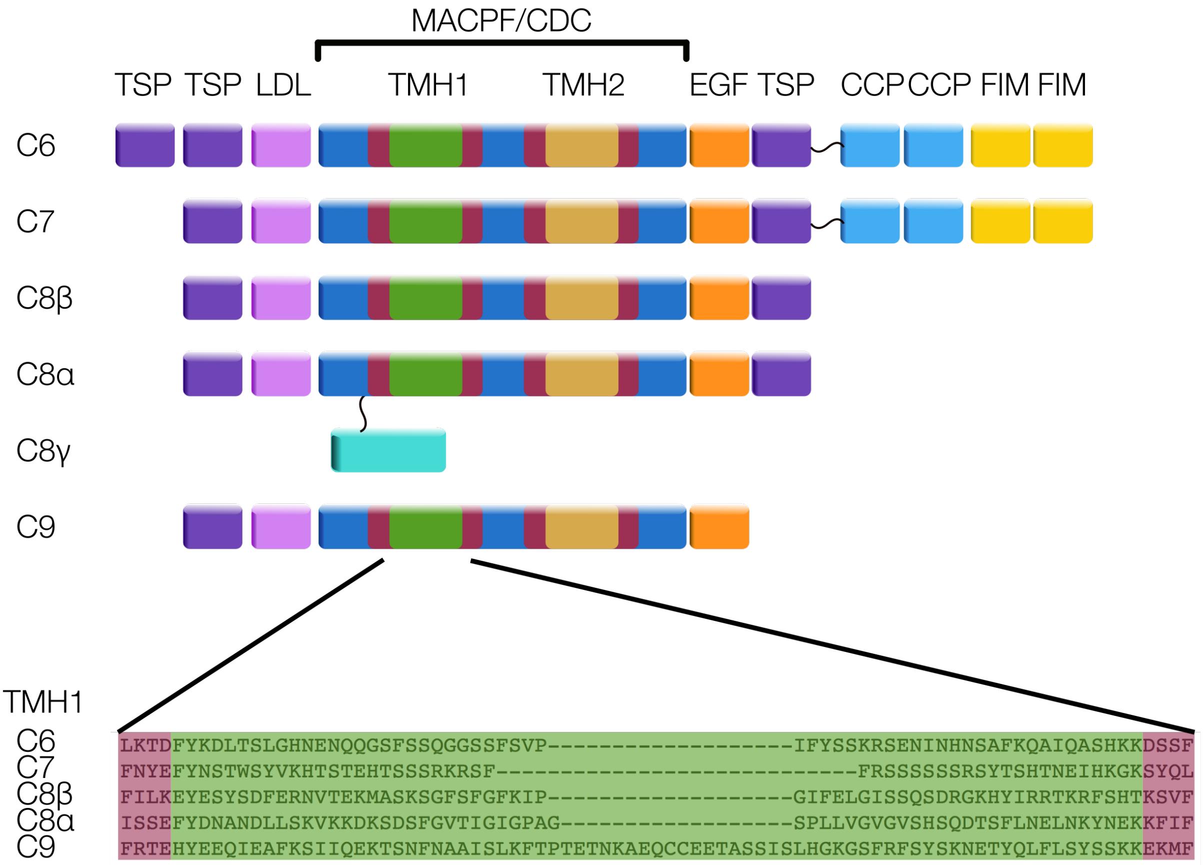

Supplementary Figure 2 Domain layout of the MAC components C6, C7, C8 and C9. These components contain a MACPF/CDC domain (blue and red) each with two transmembrane beta hairpins (TMH1, green, and TMH2 gold). The components also contain several ancillary domains, including thrombospondin-like domains (purple); low density lipoprotein receptor class A (LDL, pink); and EGF-like domain (orange); complement control protein (CCP, light blue) and factor I-membrane attack complex (FIM, bright yellow). The C8α component contains a disulphide link to C8γ (cyan). The sequence alignment of the TMH1 regions of all human protein sequences shows that this region is highly variable.

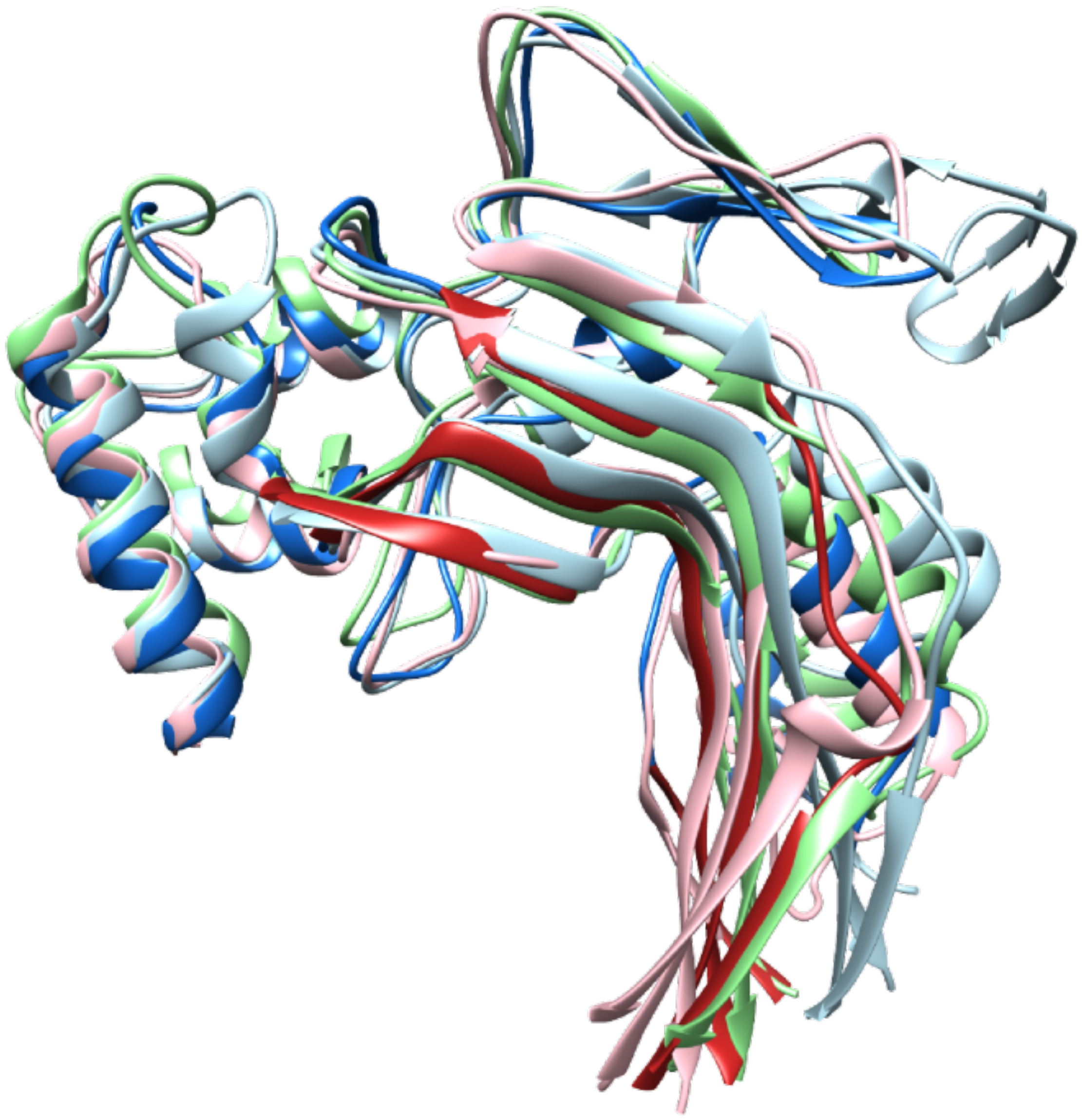

Supplementary Figure 3 Structural alignment of complement proteins. C6, pink (PDB ID 3T5O); C8α, light blue and C8β, green (PDB ID 3OJY); and C9, red and dark blue.

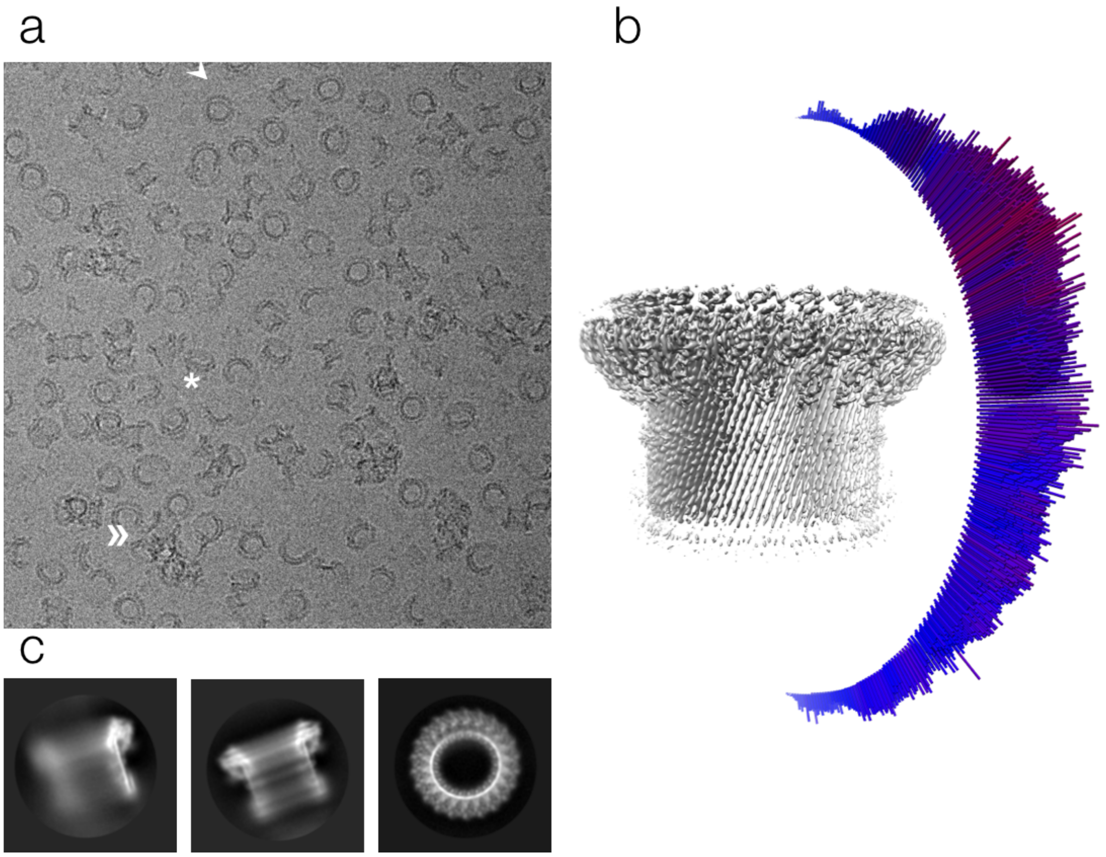

Supplementary Figure 4 Electron microscopy data of polyC9 a) Representative micrograph of vitrified recombinant human polyC9 imaged at 300 kV. Examples of a well-formed pore (arrow), an arc (asterisk) and aggregation (double-headed arrows) are shown. **b**) Angular distribution of views used in the final reconstruction. **c**) Representative 2D class averages of polyC9.

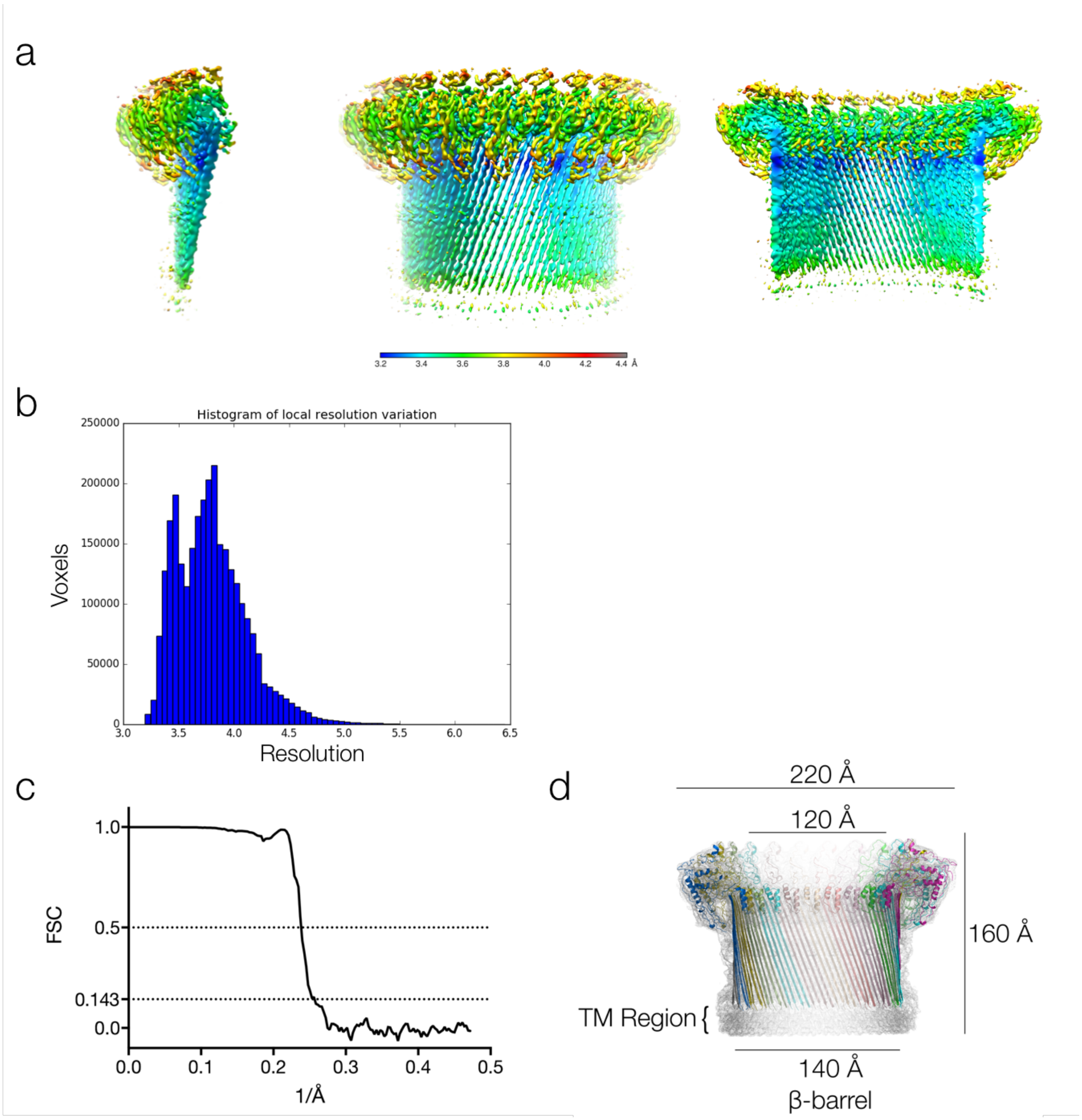

Supplemental Figure 5 Resolution data of polyC9 reconstruction a) Local variation of resolution in polyC9 reconstruction, colored according to the scale bar below, highlights local resolution features within the upper region of the β-barrel. **b**) Histogram of local resolution values throughout the reconstruction. **c**) Fourier shell correlation plot of the final polyC9 half maps. **d)** Unsharpened map with the final atomic model excluding the TM region which were not modelled due to lower resolution. The dimensions are also shown (220 Å denotes the outer-most dimension and 120 Å denotes the inner most dimension of the pore).

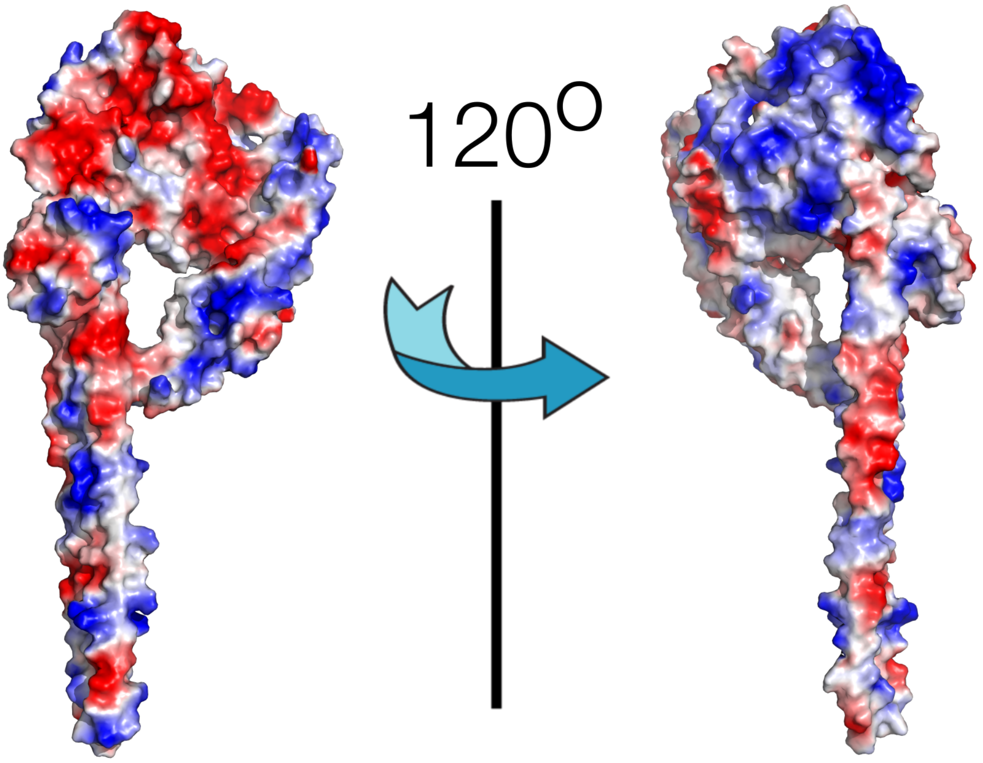

Supplementary Figure 6 Charge complementarity of the oligomer interfaces. The elongation face (left) and the binding face (right) of two neighbouring subunits of C9, rotated 120° along the y-axis. The electrostatic potential surfaces are coloured; blue, basic; red, acidic.

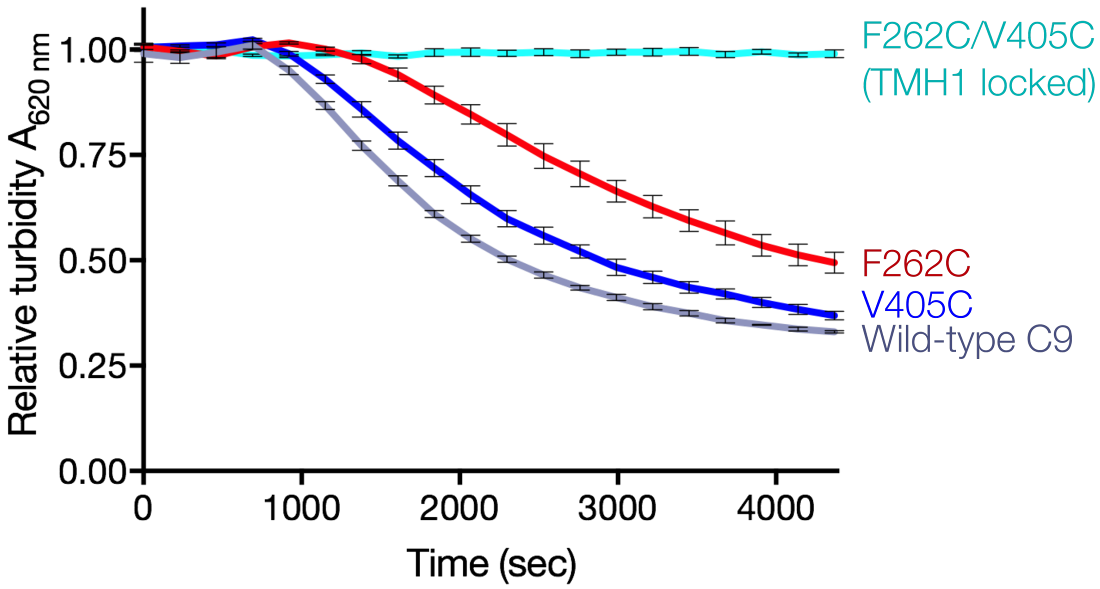

Supplementary Figure 7 Haemolytic activity of double and single cysteine mutants. The TMH1 disulphide locked C9 mutant (F262C/V405C, $C9_{mutant}$) shows a synergistic loss in haemolytic activity, as determined by the turbidity assay, against EAC1-8 compared to the single mutants (F262C and V405C); this loss in activity can be rescued by addition of a reducing agent (1 mM DDT, **Fig. 2b**). Together it suggests that the loss in activity is due to a disulphide lock. Results are reported as the average turbidity curve from three independently prepared samples (error is shown as the standard error of the mean, SEM).
